# Peptides from ‘Vaina Morada’ Black Bean Inhibit α-Amylase and α-Glucosidase: A Combined In Silico–In Vitro Study

**DOI:** 10.3390/foods14223847

**Published:** 2025-11-11

**Authors:** Filiberto Ramirez-Lozano, Jonhatan Contreras, Arturo Alfaro-Diaz, Diego Armando Luna-Vital, Anne C. Gschaedler Mathis, Judith Esmeralda Urías-Silvas, Luis Mojica

**Affiliations:** 1 Tecnología Alimentaria, Centro de Investigación y Asistencia en Tecnología y Diseño del Estado de Jalisco A.C. (CIATEJ), Guadalajara 44270, Jalisco, Mexico; firamirez_al@ciatej.edu.mx (F.R.-L.); jocontreras_al@ciatej.edu.mx (J.C.); joalfaro_al@ciatej.edu.mx (A.A.-D.); jurias@ciatej.mx (J.E.U.-S.); 2 Tecnológico de Monterrey, Instituto para la Investigación de la Obesidad, Monterrey 64849, Nuevo León, Mexico; dieluna@tec.mx; 3 Biotecnología Industrial, Centro de Investigación y Asistencia en Tecnología y Diseño del Estado de Jalisco A.C. (CIATEJ), Guadalajara 44270, Jalisco, Mexico; agschaedler@ciatej.mx

**Keywords:** antidiabetes potential, antioxidant capacity, bioactive peptides, peptidomics, molecular docking

## Abstract

The objective of this work was to evaluate the antidiabetes potential of protein hydrolysates derived from “vaina morada” black bean (*Phaseolus vulgaris* L.). Bioactive peptide sequences were identified after in silico digestion. The biological activities and molecular interactions of peptides with targeted enzymes were assayed. The degree of hydrolysis and protein profile were evaluated throughout the processing stages, including protein extraction, hydrolysis, and dialysis. Biological potential assays, including antioxidant potential (DPPH and ABTS•+), and inhibition of α-amylase and α-glucosidase enzymes, were performed. Identified bioactive peptides showed potential for inhibiting ACE and DPP-IV, as well as exhibiting antioxidant potential. Molecular docking indicated that several peptide sequences showed equal or stronger binding affinities compared to acarbose. Notably, sequence VNDNGEPTL exhibited binding energies of −10.0 kcal/mol (α-amylase) and −11.8 kcal/mol (α-glucosidase). Protein hydrolysates showed the lowest IC_50_ (113.16 µM TE/mg for ABTS•+), while dialyzed protein hydrolysates demonstrated the strongest activity for DPPH (IC_25_ of 38.83 µM TE/mg). Also, the dialyzed hydrolysate demonstrated the highest enzyme inhibition, with IC_50_ values of 0.78 mg/mL for α-amylase and 0.60 mg/mL for α-glucosidase. “Vaina morada” black bean protein hydrolysates are a rich source of multifunctional peptides, supporting their potential application in functional food formulations aimed at preventing or managing type 2 diabetes.

## 1. Introduction

In Mexico, common beans (*Phaseolus vulgaris* L.) are the most widely consumed legume, as they have been an integral part of the traditional Mexican diet since pre-Columbian civilization [1]. Mexico is recognized for its rich genetic diversity of common bean cultivars. Particularly, in the South Pacific region, encompassing the states of Guerrero, Oaxaca, and Chiapas, a large number of endemic varieties have been documented [2]. In previous work, 38 native and locally consumed cultivars of the species *P. coccineus*, *P. lunatus*, and *P. vulgaris* were collected and studied, including “vaina morada” black bean. This bean has a dark seed coat with low lightness and a medium seed size. Regarding phytochemicals, it contains high levels of anthocyanins and condensed tannins (9.42 mg cyanidin-3-glucoside equivalents (C3GE) per g of seed coat and 5.57 mg catechin equivalents (CAE) per g of seed coat on a dry basis), as well as flavonoids (18.6 mg quercetin equivalents (QE) per g of seed coat, dry basis) and total phenolics (787.5 mg gallic acid equivalents (GAE) per g of seed coat, dry basis). Among antinutritional factors, this cultivar presents low phytic acid content (24.1 mg per 100 g of flour), it also contains 3.74 g per 100 g of oligosaccharides, lectin hemagglutinating activity of 2.12 HAU (Hemagglutination Unit) per mg protein, and trypsin inhibitor content of 14.3 TIU (Trypsin Inhibitor Unit) per g flour [3].

Common beans not only serve as a major dietary source of protein in many regions but also offer a unique nutritional profile with health-promoting attributes [4,5]. In beans, proteins are found in greater abundance compared to other nutrients, representing 20 to 30% of the seed, depending on the variety [6].

In addition to their nutritional contribution, proteins can be hydrolyzed to produce protein peptides, which have been shown to exert biological potential in regulating molecular markers related to non-communicable diseases, such as hypertension, cancer, type 2 diabetes, and inflammation, among others [7]. However, the functional properties and biological activity of peptides can be influenced by factors such as cultivar and growing conditions, as well as by processing factors, including hydrolysis conditions, which are determined by the enzyme-to-substrate ratio, hydrolysis time, and protease specificity [8].

Some of the most reported proteases for hydrolyzing plant-based proteins are subtilisin, pepsin, pancreatin, papain, trypsin, and enzyme cocktail mixtures of various enzymes [9]. Moreover, emerging approaches such as subcritical water hydrolysis, microbial fermentation, and the use of commercial enzymes should be studied to produce cost-competitive bioactive protein hydrolysates on a large scale [10,11].

Common bean protein hydrolysates have shown potential for treating diabetes due to their antioxidant properties and ability to inhibit carbohydrate-degrading enzymes, such as α-amylase and α-glucosidase, as well as inhibition of dipeptidyl peptidase IV [12,13,14]. It has been reported that the consumption of common bean protein hydrolysates decreased glucose absorption and reduced oxidative stress in vivo and clinical trials [15,16].

Small peptides may modulate disease-related molecular targets. For example, Mojica described peptides (such as KTYGL) with potent bioactivity and provided mechanistic evidence, including computational docking to the enzyme’s catalytic site, that explains their inhibitory action [17]. These findings justify screening native common bean cultivars as a source of protein hydrolysates using both experimental and in silico approaches to identify candidate peptides with antidiabetic and antioxidant potential.

The research and characterization of endemic black bean cultivars is essential for identifying novel bioactive components and enhancing the nutritional and economic value of underutilized crops. The present study evaluated the antioxidant capacity and the inhibitory activity against carbohydrate-degrading enzymes of protein hydrolysates obtained from the “vaina morada” black bean, an endemic variety from Chiapas, Mexico, using in silico and in vitro approaches. It was hypothesized that enzymatic hydrolysis of this variety’s proteins would generate peptides with antioxidant and antidiabetic properties, thereby supporting their potential application in the development of functional food ingredients for managing type 2 diabetes.

## 2. Materials and Methods

### 2.1. Materials

Black bean “vaina morada” (*Phaseolus vulgaris* L.) was collected in February 2019 in the Mayan region of the state of Chiapas. Ethanol (95%), hydrochloric acid (97%), sodium chloride (95%), 2,2-diphenyl-1-picrylhydrazyl (DPPH) (>98%), 2,2′-azino-bis(3-ethylbenzothiazoline-6-sulfonic acid) (ABTS) (98%), 6-hydroxy-2,5,7,8-tetramethylchrom-2-carboxylic acid (Trolox^®^) (97%), α-amylase from porcine pancreas, α-glucosidase from *Saccharomyces cerevisiae*, acarbose (≥95%), 3,5-dinitrosalicylic acid (≥98%), p-4-nitrophenyl-α-D-glucopyranoside (≥99%), soluble starch, phosphate buffer, Folin–Ciocalteu reagent, were purchased from Sigma-Aldrich (St. Louis, MO, USA). For electrophoresis, molecular mass protein standard (10 to 250 kDa) and SafeSimplyBlue^®^ stain were obtained from Amersham Pharmacia Biotech (Carlsbad, CA, USA). Commercial HT Proteolytic^®^ and the Bio-Rad DC (Hercules, CA, USA) kit protein assays were used to quantify soluble protein.

### 2.2. In Silico and Omics Approaches

#### 2.2.1. In Silico Digestion of *Phaseolus vulgaris* L. Storage Proteins

The peptide sequences of phaseolin (PDB ID: 1PHS), arcelin (PDB ID: 5AVA), lectin (PDB ID: 1AVB), and α-amylase inhibitor (UniProt ID: LEA1_PHAVU) were retrieved from the RCSB Protein Data Bank (https://www.rcsb.org/, accessed on 5 April 2024) and UniProt (https://www.uniprot.org/, accessed on 5 April 2024) [18] databases. The biological potential of individual peptides was predicted using the BIOPEP-UWM database (https://biochemia.uwm.edu.pl/biopep-uwm/, accessed on 11 April 2024), in which subsilin was used as the protease responsible for performing in silico hydrolysis [19]. Peptide structures were designed using PepDraw (https://pepdraw.com/, accessed on 16 April 2024) [20] and MarvinSketch (v24.11, ChemAxon).

#### 2.2.2. Physicochemical Characterization of Peptides

The 97 peptides obtained from in silico digestion were evaluated using ExPASy’s ProtParam tool (https://web.expasy.org/protparam/, accessed on 19 January 2025) (peptides ≥5 amino acids) [21]. This tool determined key physicochemical properties: (1) instability index, providing a theoretical measure of peptide stability; (2) isoelectric point (pI), indicating acidic/basic nature; (3) half-life, predicting protein degradation time in food matrices; and (4) hydrophobicity index, inferring lipid/water interaction potential.

#### 2.2.3. Computational Docking

Intermolecular interactions were analyzed between peptide sequences from black bean’s storage proteins (phaseolin, arcelin, lectin, and α-amylase inhibitor). Ninety-seven peptides were designed using MarvinSketch (v24.1.1, ChemAxon) (Table 1. Molecular marker acarbose was obtained from PubChem [22] and converted to PDB format using Discovery Studio Visualizer (v21.1.0). Protein structures and selected amino acids within inhibition sites were retrieved from RCSB Protein Data Bank (https://www.rcsb.org/, accessed on 14 December 2024): α-amylase (PDB ID: 1B2Y) [23] and α-glucosidase (PDB ID: 3AJ7, *Saccharomyces cerevisiae* isomaltose), the latter being the most structurally homologous template as reported [24,25]. Docking calculations were performed with AutoDock Vina (v1.2.3), and the theoretical binding energies (kcal/mol) were calculated. For protein-peptide visualization, Discovery Studio Visualizer was used.

### 2.3. Protein Extraction

Protein extraction was performed using a methodology described by ref. [26] with modifications. Black beans were soaked in airtight bags at a 5:6 (*w*/*w*) ratio with distilled water and held at 40 °C for 4 h. After soaking, beans were dried on racks in a convection oven at 55 °C for 5 h, dehulled mechanically (discarding the hull), and the remaining cotyledon was ground and sieved through a #40 mesh (0.42 mm particle size) to enhance protein extraction. The resulting bean flour was dispersed in an aqueous medium at pH 10 (adjusted with NaOH) using a 1:10 (*w*/*w*) ratio with distilled water and stirred constantly at 40 °C for 1 h. The extract was then centrifuged at 4000 rpm and 25 °C for 20 min, and the supernatant was collected and stored at −20 °C for further hydrolysis. This protein-rich supernatant was labeled as “Concentrate” (C).

### 2.4. Enzymatic Hydrolysis

Protein hydrolysate production was carried out according to established methods [26]. The extract pH was adjusted to 7.0 before adding Proteolytic^®^ (1% *w*/*w* of soluble protein content). Hydrolysis was performed for 4 h with hourly aliquot collection. Enzyme inactivation was achieved by heating at 80 °C for 15 min, followed by centrifugation (4000 rpm, 20 min, 4 °C) to recover the supernatant (“Hydrolysate” (H)). Subsequent dialysis used a 100–500 Da membrane (Spectra/Por) with continuous stirring for 24 h, replacing distilled water every 6 h to yield the “Dialyzed Hydrolysate” (D), represents the retained fraction (>MWCO 500 Da).

The collected samples (Concentrated, Hydrolyzed, and Hydrolyzed-Dialyzed) were lyophilized and stored at −20 °C until use. Soluble protein content was determined in each sample using the Lowry (Bio-Rad, Hercules, CA, USA) method as reported by ref. [27], where 1 mg of powder was used to estimate the soluble protein purity in each corresponding sample.

### 2.5. Electrophoresis Profile Assay

The procedure was adapted from literature methods [28]. Aliquots containing 20 mg protein were prepared by combining 50 μL of each sample with 100 μL Laemmli buffer and 25 μL β-mercaptoethanol in Eppendorf tubes, followed by heat denaturation at 90 °C. Electrophoresis using 4–20% gradient SDS-PAGE gels, with samples run initially at 100 V (30 min) followed by 150 V (60 min) as needed. Gels were washed with distilled water and stained with SimplyBlue Safe Stain.

### 2.6. Degree of Hydrolysis

The degree of hydrolysis (DH) was determined following the TNBS method as previously described [29]. Briefly, total hydrolysis was performed using 6 N HCl (4.5 mL) and 500 µL of enzymatic hydrolysate solution (10 mg/mL) at 100 °C for 24 h, and the reaction was terminated with 6 N NaOH (4.5 mL). For enzymatic hydrolysis, 500 µL of the hydrolysate (10 mg/mL) was diluted in 9 mL of sodium phosphate buffer (pH 8.2). Aliquots (64 µL) were reacted with 1 mL of sodium phosphate buffer and 500 µL of 0.05% TNBS, followed by incubation at 50 °C for 30 min. The reaction was stopped by adding 1 mL of 0.1 M sodium sulfite and resting for 15 min at room temperature. Absorbance was measured at 420 nm in a spectrophotometer, and DH was calculated using the following equation: Degree of hydrolysis (%) = Hydrolysis extent / Total hydrolysis potential × 100.

### 2.7. Antioxidant Capacity Assays

Both ABTS and DPPH assays were performed using Trolox standard curves (0.01–0.275 mM) following modified literature methods [30].

#### 2.7.1. ABTS Assay

The ABTS radical cation solution was prepared by reacting 7 mM ABT with 2.45 mM potassium persulfate in the dark under constant agitation for 12 h. The solution was stored at −20 °C and diluted to an absorbance of 0.70 ± 0.02 at 734 nm. Lyophilized samples (Concentrate, Hydrolysate and Dialyzed hydrolysate) were dissolved in TBS (10 mg soluble protein/mL). Aliquots (20 μL) of samples, Trolox standards, and TBS blank were plated in triplicate on 96-well plates. After adding 180 μL of ABTS solution, absorbance was measured immediately at 734 nm. Radical scavenging activity was calculated as: % inhibition = (absorbance blank − absorbance extract)/absorbance blank × 100

#### 2.7.2. DPPH Assay

The DPPH reagent was prepared as a 60:40 (*v*/*v*) ethanol:water solution to prevent precipitation of the hydrolysate. Sample dilutions (20 μL), Trolox standards, and a TBS blank were plated in triplicate in 96-well plates. After adding 180 μL of DPPH solution, the plates were incubated in the dark for 30 min before measuring the absorbance at 517 nm. Radical scavenging activity was calculated as: % inhibition = (absorbance blank − absorbance extract)/absorbance blank × 100.

### 2.8. Biological Activity Assays

#### 2.8.1. α-Amylase Inhibition

The assay was performed using 0.02 M phosphate buffer (pH 6.9, adjusted with 1 M phosphoric acid). Enzyme solution (α-amylase in 0.02 M phosphate buffer), 1% starch solution, and DNS reagent were prepared in the same buffer. Acarbose served as the positive control.

Following modified literature methods [31], 250 μL aliquots of sample, acarbose (positive control), and phosphate buffer (negative control) were incubated at 37 °C for 5 min with agitation. After adding α-amylase solution, samples were incubated for 10 min (37 °C, agitation) followed by the addition of 1% starch solution (10 min, 37 °C). The reaction was terminated by adding DNS reagent, heating for 5 min, and then immediately cooling on ice. Absorbance was measured at 540 nm (200 μL samples in triplicate).

Equation to calculate the % inhibition: % inhibition negative = (absorbance negative control − absorbance extract)/absorbance negative control × 100 and % inhibition positive = % inhibition negative (100/% inhibition acarbose).

#### 2.8.2. α-Glucosidase Inhibition

The assay was performed as described [32] using 0.01 M phosphate buffer. Enzyme solution (1 mM α-glucosidase) and substrate (p-nitrophenyl-α-D-glucopyranoside) were prepared in buffer. Samples and acarbose control (triplicate) were incubated with the enzyme (10 min, 37 °C) before adding substrate (5 min, 37 °C). Absorbance was measured at 405 nm. Equation to calculate the % inhibition: % inhibition negative = (absorbance negative control − absorbance extract)/absorbance negative control × 100 and % inhibition positive = % inhibition negative (100/% inhibition acarbose).

### 2.9. Statistical Analysis

All assays were performed in triplicate, and the results are expressed as mean ± standard deviation. Statistical analysis was carried out using one-way ANOVA (*p* < 0.05) followed by Fisher’s LSD test for multiple comparisons among the concentrate, hydrolysate, and dialyzed hydrolysate groups (Minitab 18, State College, PA, USA). Inhibition potential is expressed as IC_50_, which represents the concentration needed to inhibit 50% of the enzyme’s activity toward the radicals. When 50% of the inhibition was not reached with the contractions used, the inhibition potential is expressed as IC_25_. Half-maximal inhibitory concentration (IC_50_) values were determined using nonlinear regression analysis (GraphPad Prism 9, San Diego, CA, USA).

## 3. Results

### 3.1. Peptide Sequencing and Predicted Biological Potential

The enzymatic hydrolysis simulation using Alcalase enabled identification of ninety-seven peptides derived from four parental storage proteins of black beans (*Phaseolus vulgaris* L.): phaseolin (4–14 amino acid peptides), lectin (4-11 amino acids), arcelin (4–10 amino acids), and α-amylase inhibitor (4–11 amino acids), dipeptides and tripeptides were excluded.

The physicochemical analysis revealed a charge distribution of 26.9% neutral, 43.3% negative, and 29.8% positive peptides, with molecular weights ranging from 0.39 kDa (TTGN from α-amylase inhibitor sequence) to 1.61 kDa (VDGHHHQQQEQQQKGS from phaseolin sequence) (Table 1). The most recurrent bioactive sequences were GH, KG, GS, QK, and DG (common in multifunctional peptides), as well as EE, QQ, and QE (associated with stimulatory and antioxidant functions in negatively charged peptides). Predicted biological activities included: (1) Angiotensin-Converting Enzyme (ACE) inhibition (most prevalent), (2) dipeptidyl peptidase-IV (DPP-IV) inhibition (linked to antidiabetes effects), and (3) antioxidant capacity (free radical neutralization).

ProtParam calculations indicated that peptides with a low isoelectric point (pI) (e.g., GINEGNTE-TETND, pI = 2.74) are more suitable for acidic food matrices, such as fermented beverages, because negatively charged residues enhance solubility and stability at low pH. In contrast, peptides with a high pI (e.g., TMNIRTHRQANS, pI = 12.48) may be better adapted to alkaline environments. However, these are less common in food systems (e.g., products with strong leavening agents or protein solutions adjusted to basic pH).

Peptides exhibiting high hydrophobicity values (e.g., EEEGQQQEEGQQQEG, 36.21 kcal mol^−1^) are predicted to interact preferentially with lipid-rich environments, suggesting potential applications in fat-containing matrices such as emulsions or concentrated dairy products. Conversely, low hydrophobicity values (e.g., APIQIW, 4.98 kcal mol^−1^) imply greater aqueous solubility, which favors their use in beverages.

Regarding the instability index, values <40 predict stable peptides, whereas values >40 indicate instability. Stable peptides may withstand thermal processing such as baking or sterilization. In contrast, highly unstable peptides (e.g., REEEES, 203.43) may be better suited for fresh or refrigerated foods, where high thermal resistance is not necessary.

Finally, estimated half-lives ranged from long (VDGHHHQQQEQQQKGS, 10–100 h; suitable for shelf-stable products) to short (RIIQL, 0.02–1 h), suggesting that some peptides may require protective strategies such as encapsulation. These findings highlight the remarkable structural and functional diversity of the identified peptides.

The theoretical binding affinity (kcal/mol) of black bean bioactive peptides against two key carbohydrate-degrading enzymes: α-amylase and α-glucosidase. The positive control, acarbose (a clinical inhibitor for both enzymes), showed affinities of −7.1 kcal/mol (α-amylase) and −8.0 kcal/mol (α-glucosidase). The peptide with the highest affinity for α-amylase was VNDNGEPTL (−10.0 kcal/mol), surpassing acarbose, while NKIL exhibited the lowest affinity (−4.0 kcal/mol). Other high-affinity peptides included NQIEIDMNS (−9.7 kcal/mol), APIQIW (−9.4 kcal/mol), NKPDDPEAHI (−9.3 kcal/mol), and VNNPQIHEF (−9.3 kcal/mol). For α-glucosidase, VNDNGEPTL also showed the highest affinity (−11.8 kcal/mol), significantly outperforming acarbose. Other notable peptides were VHDY (−10.3 kcal/mol), GTKCNF (−10.2 kcal/mol), IIDAF (−10.0 kcal/mol), and VNNPQIHEF (−10.0 kcal/mol), with NKIL again showing the lowest affinity (−4.4 kcal/mol).

#### Computational Docking

**Figure 1:**
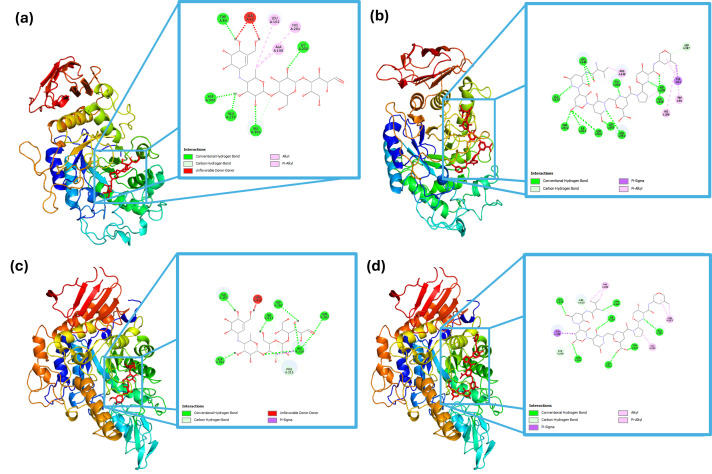
Molecular interactions of α-amylase with (**a**) acarbose and (**b**) peptide (VNDNGEPTL); and α-glucosidase with (**c**) acarbose and (**d**) peptide (VNDNGEPTL).

Figure 1 shows the detailed molecular interaction of the enzymes with the control (acarbose) and the peptide with the strongest binding (VNDNGEPTL). In panels (a) and (b), the interaction of α-amylase with acarbose and the peptide, respectively, is shown at the active site. A detailed view reveals that acarbose formed conventional hydrogen bonds with Trp59, Lys200, Glu233, Gly306, and Asp300, as well as hydrophobic interactions with Leu162, Ala198, and His201. Meanwhile, the peptide formed hydrogen bonds with Asp197, Asn301, Gly304, His305, Gly306, Ala310, Ile312, Thr314, Arg346, Asn352, and Asp353, hydrophobic interactions with Trp58, Phe348, and His299, and a non-covalent bond with Tyr62.

In Figure 1c,d, the interaction of α-glucosidase with acarbose and the peptide sequence at the catalytic site is depicted. Acarbose formed hydrogen bonds with Tyr158, Asp242, His280, Asp307, Ser311, and Pro312, whereas the peptide formed hydrogen bond with Ser240, Ser241, His280, Thr310, Ser311, Arg315, glu332, Asn415, and Arg442, hydrophobic interactions with Phe308, Ala329, and Arg335, and a non-covalent bond with Tyr153.

Molecular docking results indicated that black bean protein hydrolysates may contain peptide sequences with high affinity toward carbohydrate-hydrolyzing enzymes, supporting their potential role in postprandial glycemic control. Notably, VNDNGEPTL exhibited a predicted binding energy of −11.8 kcal mol^−1^ against α-glucosidase, surpassing that of acarbose, a clinically validated α-glucosidase inhibitor. A strong interaction was also observed with α-amylase (−10.0 kcal mol^−1^).

Although the docking poses revealed multiple hydrogen bonds and hydrophobic interactions, the analysis did not explicitly confirm binding to catalytic residues such as Asp197, Glu233, and Asp300 in α-amylase or Asp518 and Asp616 in α-glucosidase, which are well documented as essential for glycosidic bond hydrolysis and are common targets of carbohydrate-mimicking inhibitors. The absence of direct annotation limits the ability to classify the inhibition mechanism as strictly competitive. Nevertheless, the peptide’s proximity to the active site and the observed binding energies suggest interference with enzymatic function, consistent with either competitive or mixed-type inhibition.

These findings align with previous reports on legume-derived peptides that inhibit carbohydrate-digesting enzymes through interactions with conserved domains [33]. The dual-inhibition profile observed for VNDNGEPTL highlights its potential as a multitarget candidate for functional foods or nutraceuticals intended for the management of type 2 diabetes. Further validation by molecular dynamics simulations and kinetic assays is recommended to substantiate these preliminary observations.

### 3.2. Protein Hydrolysate Characterization

#### 3.2.1. Protein Concentration in the Hydrolysate Obtention Process

**Figure 2:**
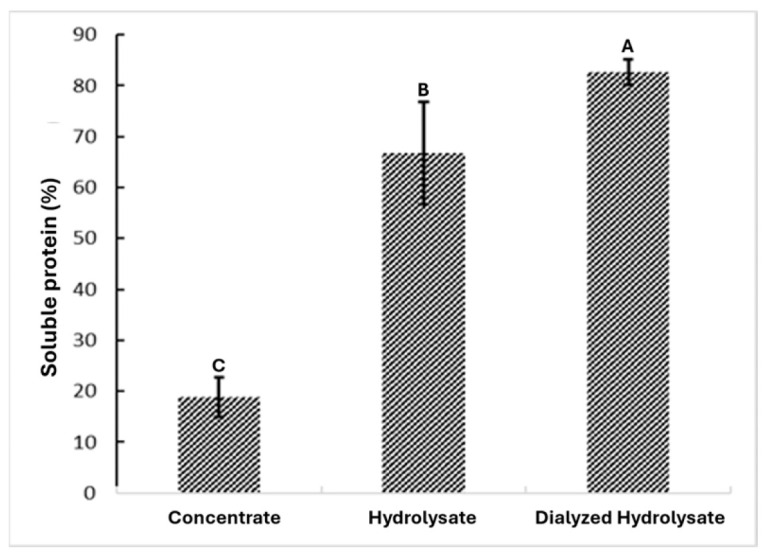
Concentration of soluble protein at the end of the extraction, hydrolysis, and dialysis processes. Different letters indicate significant differences (*p* < 0.05).

The extracts from each processing stage demonstrated varying purity levels, as shown in Figure 2. The sample labeled “Concentrated” (obtained through alkaline protein extraction) showed 18.82% percentage of soluble protein, while the “Hydrolyzed” reached 66.8% percentage of soluble protein, and the “Dialyzed Hydrolyzed” achieved 82.65% percentage of soluble protein. Dialysis after enzymatic hydrolysis is a crucial purification step, as it removes low-molecular-weight components (salts, soluble sugars, small metabolites, and peptides with molecular weights below the chosen MWCO, in this case 500 Da), thereby reducing ionic strength and matrix interferences that can affect small components. This confirms that each stage effectively increases the purity of black bean protein.

#### 3.2.2. Protein Profile

**Figure 3:**
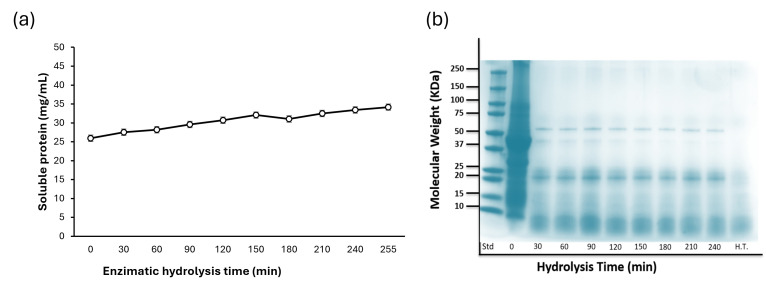
(**a**) Enzymatic hydrolysis of bean (*Phaseolus vulgaris* L.) protein isolates using HT Proteolytic^®^. (**b**) The protein profile by SDS-PAGE of the protein hydrolysates of purple podded black bean.

The enzymatic hydrolysis process showed an increase in soluble protein, starting from an initial concentration of 25.9 mg SP/mL. At 30 min post-hydrolysis initiation, the soluble protein content was quantified at 27.52 mg PS/mL, showing a consistent increase of approximately 1 mg SP/mL per 30-minute interval until reaching 32.07 mg SP/mL at 150 min (Figure 3a). Beyond this point, the soluble protein concentration remained stable, with only a minor increase to 34.17 mg SP/mL by the end of the hydrolysis process. The recovery percentage ranged between 59.7% and 73.9% of dry protein, while dialysis resulted in a dry protein content of 80.9% to 84.4% in the extract.

Electrophoresis (Figure 3b) showed the protein fraction profile during the hydrolysis process. At time zero, the predominant protein bands corresponded to well-documented common bean proteins, including 9S lipoxygenase (≈approximately 100 kDa), legumin (≈approximately 75 kDa), phaseolin (50–37 kDa), lectin (≈approximately 25 kDa), α-amylase inhibitor (≈approximately 15 kDa), and protease inhibitor (≈approximately 10 kDa).

After 30 min of enzymatic hydrolysis, marked changes in the banding pattern were observed, and only phaseolin, lectin, arcelin (50–25 kDa), α-amylase inhibitor (≈approximately 15 kDa), and protease inhibitor (≈approximately 10 kDa) remained detectable. Although these proteins were still present at subsequent time points, progressive degradation was evident. After 4 h of enzymatic treatment, the bands corresponding to phaseolin and other residual proteins had gradually faded, indicating advanced hydrolysis of the major fractions.

#### 3.2.3. Degree of Hydrolysis

**Figure 4:**
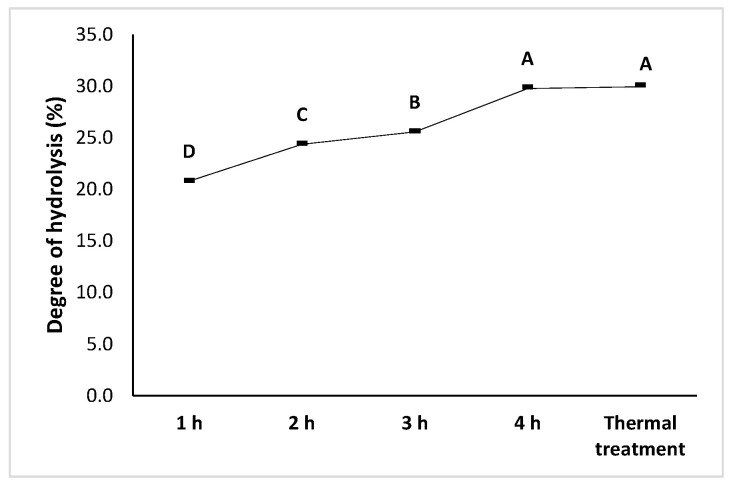
Degree of hydrolysis of black bean (*Phaseolus vulgaris* L.) protein isolates. Different letters indicate significant differences (*p* < 0.05).

The progression of enzymatic hydrolysis was monitored by the degree of hydrolysis (DH) (Figure 4). A gradual increase in DH was observed from 1 to 4 h of digestion with HT Proteolytic^®^ at a constant concentration, ranging from 20.8% (1 h) to 29.9% (4 h) (*p* < 0.05).

The degree of hydrolysis increased from 60 to 240 min, indicating an initial phase of rapid cleavage of accessible peptide bonds. Beyond this point, DH tends to maintain, due to substrate depletion, enzyme inhibition by hydrolysis products, or reduced accessibility of cleavage sites within the protein matrix. These observations are consistent with the previously reported hydrolysis kinetics for legume protein isolates.

Overall, the hydrolysis kinetics observed demonstrate that the proteolytic system employed was effective in progressively degrading black bean proteins, thereby releasing low-molecular-weight peptides that will subsequently be characterized for their bioactivity.

### 3.3. Biological Potential

#### 3.3.1. Antioxidant Potential

**Figure 5:**
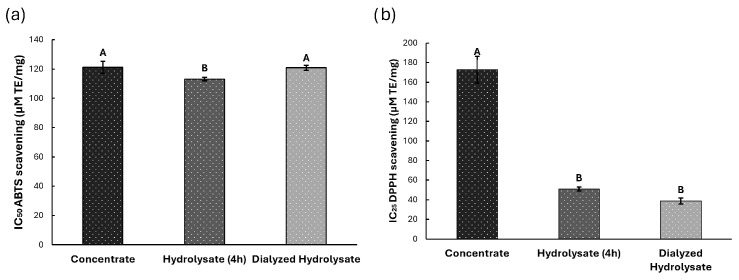
Antioxidant potential for Concentrate, Hydrolyzed and Dialyzed Hydrolysate extracts. (**a**) ABTS-+ free radical. (**b**) DPPH free radical. Different letters indicate significant differences (*p* < 0.05).

The ABTS+ radical scavenging capacity of the samples was evaluated. The results, expressed as IC_50_ values in Trolox equivalents (TE), revealed that the hydrolyzed extract (113.16 ± 1.24 µm TE/mg) showed comparable activity to both concentrated (121.20 ± 4.13 µm TE/mg) and dialyzed (120.83 ± 1.65 µm TE/mg) extracts, with no statistically significant differences (*p* > 0.05) (Figure 5a).

DPPH radical scavenging activity (Figure 5b), measured at IC_25_, demonstrated significant differences (*p* < 0.05) among extracts. The dialyzed extract exhibited the highest antioxidant activity (38.83 ± 3.02 µm TE/mg), followed by the hydrolysate (50.91 ± 2.16 µm TE/mg) and concentrated extract (172.72 ± 13.55 µm TE/mg).

#### 3.3.2. Antidiabetes Potential

**Figure 6:**
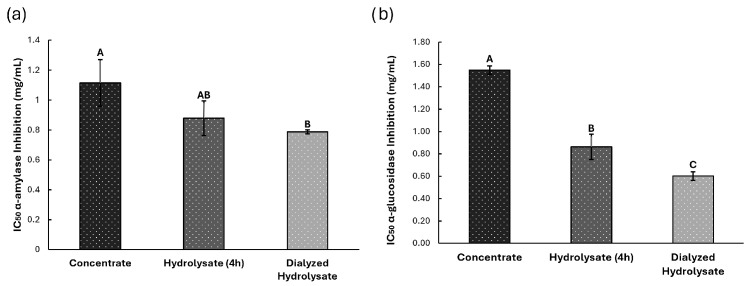
Antidiabetic potential, (**a**) Inhibition of α-amylase enzyme and (**b**) Inhibition of α-glucosidase enzyme, for Concentrate, Hydrolyzed and Dialyzed Hydrolysate extracts. Different letters indicate significant differences (*p* < 0.05).

The inhibitory potential of black bean protein hydrolysates against α-amylase and α-glucosidase was evaluated by enzymatic assays, and the results were expressed as IC_50_ values (Figure 6). For α-amylase inhibition (Figure 6a), the dialyzed extract (0.78 ± 0.01 mg/mL) and the hydrolyzed extract (0.87 ± 0.11 mg/mL) exhibited comparable activity, both significantly stronger compared to concentrated extract (1.11 ± 0.15 mg/mL) (*p* < 0.05).

A similar trend was observed in α-glucosidase inhibition assays (Figure 6b). The dialyzed extract (0.60 ± 0.03 mg/mL) and the hydrolyzed extract (0.86 ± 0.11 mg/mL) differ significantly from each other. Yet, both showed significantly greater inhibition potential compared to the concentrated extract (1.55 ± 0.03 mg/mL) (*p* < 0.05).

## 4. Discussion

### 4.1. Computational Modeling of Bioactive Peptide Interactions in Food Matrices: Molecular Docking and Stability Analysis

The instability index was employed to estimate the structural stability of peptides, with values below 40 indicating that the peptide is likely to remain stable under typical processing and storage conditions relevant to functional foods. The predicted half-life provides an estimate of peptide persistence in biological systems or food matrices. Additionally, peptide half-life analysis provides an approximation of protein degradation times in various biological environments. Table 1 illustrates contrasting examples: peptides such as GINANNNNRNL (instability index ≈ 12.6; half-life ≈ 10–30 h) exhibit a favorable profile for formulations requiring extended shelf life, whereas sequences such as EEEGQQEEGQQEG (instability index ≈ 143.7; half-life ≈ 1–10 h) are theoretically unstable and would require protective strategies (e.g., encapsulation) for practical application [34,35].

Numerous studies have confirmed that legume-derived peptides can inhibit the activity of α-amylase and α-glucosidase. For instance, in a soybean study, two octapeptides were identified (LDQTPRVF and SRNPIYSN) that exhibited theoretical binding energies of −9.1/−8.7 kcal·mol^−1^ (amylase/glucosidase) for LDQTPRVF peptide sequence and −7.6/−7.5 kcal·mol^−1^ for SRNPIYSN peptide sequence [36]. These interactions were stabilized through multiple hydrogen bonds with catalytic residues (e.g., Thr163, His201, Asp300 in amylase) and π-π stacking (e.g., Phe151) [37]. Notably, the peptide VNDNGEPTL, referenced in Table 1, demonstrated stronger affinities (−10.0 and −11.8 kcal·mol^−1^) than previously reported peptides, suggesting that sequence variants may further enhance binding. Detailed docking analysis revealed that LDQTPRVF binds outside the glucosidase active pocket (non-competitive inhibition) [38], while SRNPIYSN competes for the entrance of the active site. Collectively, these findings indicate a consistent binding mechanism, in which N-terminal residues (Leu, Ser) and hydrophobic C-terminal residues (Phe) frequently form additional enzyme interactions, thereby increasing complex stability [39,40].

Peptides from the “Vaina Morada” black bean exhibit more favorable theoretical binding affinities than those reported for soybean and chickpea peptides. In soybean, Tang et al. [41] (AutoDock Vina) identified two octapeptides with dual affinity LDQTPRVF (−9.1/−8.7 kcal mol^−1^ for α-amylase/α-glucosidase) and SRNPIYSN (−7.6/−7.5 kcal mol^−1^) and, after synthesis, confirmed inhibitory activity with IC_50_ values in the millimolar range, thereby validating the in silico predictions. In chickpea, Chandrasekaran et al. [42] (AutoDock Vina) reported SPQSPPFATPLW as the top binder to α-amylase (−8.4 kcal mol^−1^) and YVDGSGTPLT to α-glucosidase (−7.3 kcal mol^−1^); after bromelain digestion, the best were KMTAGSGVT (−7.1 kcal mol^−1^, α-amylase) and GLTQGASLAGSGAPSPLF (−6.5 kcal mol^−1^, α-glucosidase). Quintero-Soto et al. [43] further demonstrated dual in vitro inhibition by the tetrapeptide FGKG (54% for α-amylase, 56% for α-glucosidase), supported by docking studies at the enzymes’ catalytic sites. Vaina Morada peptide VNDNGEPTL shows more negative binding energies, indicating a stronger potential to block the enzyme.

In summary, legume hydrolysates produce peptides with a measurable affinity for α-amylase and α-glucosidase. Although they are less potent than pharmacological agents, they offer safety advantages, such as serving as dietary adjuncts. Research on soybeans concluded that these molecules “do not match drug effects but can serve as long-term functional food adjuncts for diabetes management.” [44,45,46]. Indeed, legume hydrolysates (with safe nutritional profiles) represent promising candidates for the chronic management of hyperglycemia (with no acute toxicity up to >5000 mg/kg). Overall, the literature confirms that legume-derived peptides share mechanisms and inhibitory activity comparable to those of the originally reported examples (e.g., VNDNGEPTL), supporting their feasibility as functional food ingredients for the prevention of type 2 diabetes.

### 4.2. Protein Hydrolysate Characterization and Biological Potential: Correlating Hydrolysis Efficiency, Antioxidant Activity, and α-Amylase/α-Glucosidase Inhibition in Black Bean “Vaina Morada” Peptides

The protein banding patterns matched those previously reported by Santamaria, L. et al. (2024) [47], particularly the 15–25 kDa pepsin-resistant band, suggesting the presence of glutamic acid-rich peptide regions. The size variations observed under different digestion conditions may be attributed to glycosylation or sugar modifications, thereby enhancing food applicability [48].

Although enzymatic hydrolysis was performed using commercial Alcalase, other studies have employed enzyme mixtures (Flavourzyme/Alcalase/Neutrase) to enhance peptide bioactivity [49] or conducted comparative studies of Alcalase versus papain [50]. Nevertheless, in this study, specific peptides generated after Alcalase hydrolysis demonstrated both antioxidant and inhibitory activity at relatively low concentrations. These findings underscore the importance of sequenced peptides and suggest that using enzyme combinations could further enhance the bioactive properties of black bean protein hydrolysates.

The degree of hydrolysis indicates the characteristics of the hydrolysate by showing the extent to which peptide bonds have been cleaved. While [15] reported higher hydrolysis rates with pepsin (27.7%) compared to Alcalase (23.6%) after 360 min, commercial Alcalase reached 29.9% after 240 min in this work. Authors reported [51] that a enzyme-substrate concentrations of 7% improve tthe he degree of hydrolysis in abalone protein.

Legume hydrolysates also exhibit significant antioxidant activity measured by DPPH and ABTS assays. For example, bromelain-hydrolyzed black bean showed potent activity: the <3 kDa fraction required only 0.1 mg/mL to scavenge 50% DPPH radicals (IC_50_ = 100 µg/mL) and 0.16 mg/mL for ABTS (IC_50_ = 160 µg/mL) [52]. These relatively low values (high activity) compare favorably with many food peptides. In contrast, chickpea hydrolysates achieved 70–90% ABTS inhibition with IC_50_ between 1.54 and 2.12 mg/mL [53]. Soybean showed intermediate values: nanocomposite hydrolysis produced peptides with DPPH IC_50_ = 0.553 mg/mL and ABTS = 0.002 mg/, surpassing glutathione used as a control [41]. Generally, legume peptide hydrolysates exhibit antioxidant capacities comparable to those of chemical standards (Trolox) or other natural compounds. Although reporting units vary (IC_50_ mg/mL or µmol TE/mg protein), several protein hydrolysates effectively scavenge radicals. For example, dialyzed hydrolysate showed DPPH IC_25_ of 38.83 µM TE/mg, while pea and black bean peptides clearly exerted this activity at lower concentrations [54].

These antioxidant effects are particularly relevant in diabetes, where oxidative stress exacerbates inflammation and insulin resistance. In vitro and in vivo studies suggest peptide radical neutralization may improve cellular redox status and protect pancreatic β-cells. For example, in H_2_O_2_-exposed human hepatocytes, pea peptides attenuated ROS production and increased antioxidant enzyme activity [55]. Thus, incorporating these hydrolysates into functional foods could mitigate oxidative markers. Researchers noted that chickpea peptides have “high antioxidant potential” and could serve as nutraceutical ingredients against oxidative stress-related diseases and diabetes [43]. Similarly, black bean peptides prolonged the stability of sunflower oil in Rancimat tests, demonstrating protection against lipid oxidation [56].

Regarding the in vitro inhibitory potential for carbohydrate-degrading enzymes, soybean peptides showed relatively high IC_50_ values reported in mM units. LDQTPRVF inhibited α-amylase with an IC_50_ of 3.08 mM and α-glucosidase with an IC_50_ of 2.52. These values are substantially higher compared to acarbose (IC_50_ of 0.45 mM for α-amylase and 0.0608 mM for α-glucosidase) [57]. However, other grain protein hydrolysates showed comparable potency: fermented rice bran reported inhibition with IC_50_ values of 8.59 mM (α-glucosidase) and 2.58 mM (α-amylase). Moreover, *Phaseolus lunatus* (lima bean) protein hydrolysates achieved α-glucosidase IC_50_ of 0.86 mg/mL (similar to our baseline 0.60 mg/mL) [47]. The legume fractions < 1 kDa (*P. lunatus* and *P. vulgaris*) reduced postprandial glycemia in vivo at low doses. They showed in vitro IC_50_ in 0.86–0.75 mg/mL range [58]. Purified chickpea peptide mixtures demonstrated 50% inhibition of α-amylase and α-glucosidase, confirming their potential as antidiabetes agents [59].

Vaina Morada bean exhibited strong enzymatic inhibition potential (IC_50_ 0.78 mg·mL for α-amylase and 0.60 mg·mL for α-glucosidase), and antioxidant activity measured by ABTS (IC_50_ 113 µM TE·mg) and DPPH (IC_25_ 38.83 µM TE·mg), these values surpassing those of other varieties under comparable conditions. In Carioca common bean, Ohara et al. [48] reported that protein hydrolysates reached 50.96% DPPH (10 mg·mL), 100% inhibition of α-amylase, and 35% inhibition of α-glucosidase depending on the protease blend. In black turtle bean [60], ethanolic extracts showed an IC_50_ of 2.60 ± 0.61 mg·mL against α-glucosidase. In kidney beans (white/red), an intermediate potential was observed for ABTS and α-glucosidase inhibition, whereas in red kidney the DPPH values (IC_50_ 28.1–76.1 mg·mL) and α-amylase inhibition (IC_50_ 40 mg·mL) which were less effective compared to Vaina Morada bean.

From commercial and regulatory perspectives, these findings create opportunities. Legumes are widely consumed, which facilitates their recognition as safe. Some legume hydrolysates are already used (white bean extracts as carbohydrate blockers). However, for claims related to “postprandial health” or “oxidative stress reduction,” legislation requires clinical evidence. To date, no authorized specific claims exist for legume peptides; in the EU, health claims require controlled human trials [61]. The nutraceutical industry could capitalize on this: peptide hydrolysates or fractions could be formulated into capsules or fortified foods targeting individuals with diabetes or those concerned about oxidative aging. However, clinical studies demonstrating effect plus stability, bioavailability, and allergenicity assessments will be required [62].

The literature indicates that legume bioactive peptides have dual functionality: moderate enzymatic inhibition (promising for glycemic control) and notable antioxidant activity. While not rivaling medications in potency, their high safety profile makes them useful as long-term functional ingredients or nutraceuticals for preventive use [63]. With the growing demand for natural products to manage type 2 diabetes and oxidative stress, these hydrolysates offer significant commercial potential. Still, success will require better molecular characterization, proper formulation, and clarification of the regulatory framework, supported by studies that substantiate the glycemic benefits.

## 5. Conclusions

The results confirm the hypothesis that protein hydrolysates from “vaina morada” black bean generate bioactive peptides capable of effectively inhibiting key carbohydrate-digesting enzymes and neutralizing free radicals. The peptide VNDNGEPTL stands out as it surpassed acarbose in binding affinity for both α-amylase and α-glucosidase. At the same time, dialyzed hydrolysates showed higher efficacy in antioxidant assays (ABTS and DPPH) and antidiabetes assays (inhibition of α-amylase and α-glucosidase enzymes). This work highlights their applicability as functional ingredients in acid-pH functional beverages, as well as in bakery products and nutritional supplements for the prevention of type 2 diabetes, due to their peptide solubility. Future research should explore the use of enzyme combinations and peptide encapsulation to protect short half-life peptides during processing and digestion, as well as in vivo evaluations and clinical trials to validate biological efficacy and determine the optimal dose. These findings enhance the potential of Mexican endemic black bean varieties, promoting their use in the development of functional food products.

## Figures and Tables

**Table 1:** Characteristics of peptides obtained from black bean hydrolysis observed in silico.

Parental Protein	Peptide Sequence	Net Charge	Mass (Da)	Bioactive Sequence	Biological Potential	Isoelectric Point (Ip)	Hydrophobicity (Kcal × mol^−1^)	Instability Index	Estimated Half-Life (h)	Affinity (kcal/mol)	
										α-Amylase	α-Glucosidase
Phaseolin	VDGHHHQQEQQKGS	−1	1613.7274	GH, KG, GS, QK, DG, HH, GHH, HHH, QE, QQ, VD	ACE, Antioxidative, DPP-IV	6.35	30.34	47.51	10–100	−8.2	−7.0
	EEEGQQEEGQQEG	−6	1475.5628	GQ, EG, EEE, EE, QE, QQ	ACE, Stimulating, Neuropeptide, DPP-IV	2.76	36.21	143.72	1–10	−6.7	−8.3
	HQQEQQKGRKGAF	2	1540.7837	AF, GA, GR, KG, QK, GA, QE, QQ, RK	ACE, DPP-IV	10.58	25.44	48.9	3.5–10	−9.1	−8.2
	GINANNNNRNL	1	1212.5943	GI, IN, NA, NL, NN, NR, RN	ACE, DPP-IV, Renin inhibitor	11.13	14.09	12.59	10–30	−8.7	−9.7
	DNQKIPAGTIF	0	1202.6276	IPA, IP, IF, AG, GT, QK, PA, DN, KI, NQ, TI	ACE, DPP-IV	6.77	14.05	1.69	1.1–10	−8.1	−9.9
	VGPKGNKETL	1	1041.5801	GP, VG, KG, NK, KE, VGP, ET, PK, TL	Antiamnestic, ACE, DPP-IV, Antithrombotic, Regulating	9.93	18.96	7.21	10–100	−8.4	−7.8
	KQDNTIGNEF	−1	1164.5394	IG, EF, DN, NE, NT, QD, TI	ACE, DPP-IV, Renin inhibitor, CaMPDE, Hypolipidemic	4	19.01	34.96	0.03–1.3	−7.4	−9.1
	VNNPQIHEF	−1	1096.5286	PQ, EF, NP, HE, IH, NN, QI, VN	ACE, DPP-IV, Renin, CaMPDE, Hypolipidemic, DPP-III	5.06	13.18	40.8	10–100	−9.3	−10
	VNPDPKEDL	−2	1025.5013	KE, VNP, DL, NP, KE, PK, VN	ACE, DPP-IV	3.69	21.03	18.71	10–100	−8.8	−8.9
	VKPDDRREY	0	1176.587	VK, RR, EY, KP, VKP, DR	ACE, Antibacterial, Neuropeptide, Antioxidative, Haemolytic, Antiviral, DPP-IV, Leucyltransferase	6.95	24.2	44.2	10–100	−8.3	−9.1
	PQQADAEL	−2	870.407	DA, PQ, AEL, EL, AD, AE, QA, QQ	ACE, Antioxidative, DPP-IV, α-glucosidase, DPP-III	2.98	16.6	46.29	20	−7.7	−9.0
	TQGDNPIF	−1	890.4121	IF, QG, GD, TQ, NP, DN, PI	ACE, DPP-IV	3.05	11.87	−4.28	7.2–20	−8.4	−9.2
	IEMKEGAL	−1	889.4564	GA, EG, IE, KE, AL, MK	ACE, DPP-IV	4.09	16.57	53.06	0.3–20	−6.8	−7.6
	VNEGEAH	−2	754.3236	GE, EG, EA, AH, NE, VN	ACE, Antioxidative, DPP-IV, alpha-glucosidase, DPP-III	4.07	19.53	−14.33	10–100	−6.7	−7.8
	TERTDNS	−1	821.3504	TE, ER, DN, TD	ACE, DPP-IV	4	18.79	36.09	7.2–20	−7.8	−8.9
	VAIKATS	1	688.4107	AI, KA, VA, AT, TS	ACE, DPP-IV, DPP-III	10.14	10.83	−3.56	10–100	−6.2	−7.8
	REEEES	−3	777.313	EEE, EE, ES	Stimulating, DPP-IV	3.62	24.69	203.43	0.02–1	−7.0	−7.8
	TEAQQS	−1	662.2862	EA, TE, QQ, QS	ACE, DPP-IV, Alpha-glucosidase	3.13	14.28	145.77	7.2–20	−5.8	−6.8
	EQIEEL	−3	759.3638	IE, EE, EL, QI	ACE, Stimulating, Antioxidative, DPP-IV	2.93	17.19	168	0.3–10	−7.2	−7.7
	VIPAAY	0	632.3523	IPA, AY, IP, AA, PA, VI	ACE, Antioxidative, Hypotensive, DPP-IV	5.45	6.75	35.63	10–100	−8.5	−9.7
	AGKTDN	0	604.2808	AG, GK, DN, KT, TD	ACE, DPP-IV	6.76	17.09	−5.82	4.4–20	−7.3	−8.4
	MMRAR	2	663.33	RA, AR, MM, MR	ACE, Antioxidative, AU-MP, DPP-IV	12.49	10.68	−12.84	10–30	−6.0	−7.1
	QDNPF	−1	619.2594	NP, DN, PF, QD	DPP-IV, ACE2	3.05	11.59	79.28	0.1–10	−7.8	−9.1
	KPETL	0	586.3316	KP, ET, TL, PE	ACE, Antioxidative, DPP-IV, alpha-glucosidase, DPP-III	6.53	13.47	27.68	0.03–1.3	−6.1	−7.2
	RIIQL	1	641.4213	II, IQ, QL, RI	Stimulating, DPP-IV	10.73	6.99	8	0.02–1	−7.6	−8.5
	EEINR	−1	659.3229	EI, EE, IN, NR	ACE, Stimulating, DPP-IV, Renin	4.08	16.7	111.72	0.3–10	−7.6	−8.3
	VNIDS	−1	546.2641	VN	DPP-IV	3.05	11.27	134.4	10–100	−7.7	−9.0
	KHAKS	2	569.3277	HA, KH, KS	DPP-IV	10.57	16.79	8	0.03–1.3	−5.9	−7.3
	IGRAL	1	528.3375	RA, IG, GR, AL	ACE, AUMP, DPP-IV	11.12	8.99	8	0.3–20	−6.9	−7.7
	INKQS	1	588.3222	NK, IN, QS	ACE, DPP-IV	10.15	11.66	190.6	0.3–20	−6.7	−7.4
	KNQY	1	551.2696	NQ, QY	DPP-IV	9.48	11.61	-	-	−6.7	−7.8
	GHIR	1	481.2755	IR, HIR, GH, HI	ACE, Antioxidative, DPP-IV, CaMPDE, Renin	11.13	12.07	-	-	−5.8	−7.4
	DQQS	−1	476.1861	DQ, QQ, QS	DPP-IV	3.05	13.54	-	-	−5.7	−7.2
	KHIL	1	509.3317	IL, HI, KH	ACE, Stimulating, Neuropeptide, DPP-IV	9.8	10.66	-	-	−5.9	−6.5
	VPHY	0	514.2533	HY, VP, PH, PHY	ACE, Antioxidative, Anti-inflammatory, DPP-IV	7.8	9.2	-	-	−8.7	−8.7
	DGKD	−1	433.1803	GK, DG, KD	ACE, Antioxidative	3.91	19.13	-	-	−6.4	−7.4
	VMKL	1	489.2976	KL, VM, MK	ACE, DPP-IV	10.14	8.32	-	-	−6.2	−7.5
	RAEL	0	487.2747	RA, AEL, EL, AE	ACE, Antioxidative, AUMP, DPP-IV	6.51	12.59	-	-	−6.5	−8.3
Lectin	GINEGNTETND	−3	1162.4722	GI, EG, TE, ET, IN, ND, NE, TN, NT	ACE, DPP-IV	2.74	23.03	−10.22	10–30	−9.0	−9.8
	DPKQRHIGID	0	1177.6186	IG, GI, RHI, DP, HI, PK, RH	ACE, Antioxidative, DPP-IV	7.92	21.94	87.39	0.03–10	−8.7	−9.2
	VNDNGEPTL	−2	957.4389	GEP, GE, NG, PT, EP, DN, ND, TL, VN	ACE, DPP-IV, DPP-III	2.98	16.7	5.21	10–100	−10.0	−11.8
	QPKTNAGL	1	827.4488	GL, AG, QP, KT, NA, PK, TN	ACE, DPP-IV	9.84	13.11	14.04	0.1–10	−8.2	−8.6
	VNGENAE	−2	731.3076	GE, NG, AE, NA, VN	ACE, DPP-IV, DPP-III	2.92	18.05	−23.67	10–100	−7.4	−9.7
	APIQIW	0	726.4053	IW, AP, IQ, PI, QI	ACE, DPP-IV	5.71	4.98	40.43	4.4–20	−9.4	−9.9
	DNTTGA	−1	577.2336	GA, TG, DN, NT, TT	ACE, DPP-IV	3.13	14.54	−34.12	0.03–10	−8.1	−8.9
	GPADGL	−1	528.2536	GP, GPA, GL, DG, DGL, PA, AD	Antiamnestic, ACE, DPP-IV, Antithrombotic, Regulating, Alpha-glucosidase	3.12	13.23	26.28	10–30	−7.3	−7.7
	DTCINL	−1	677.3044	IN, NL	DPP-IV	3.12	10.25	−16.72	0.03–10	−7.3	−8.3
	IKTTPW	1	744.4158	TP, PW, KT, TT	ACE, Antioxidative, DPP-IV	10.15	8.13	−10.62	0.3–20	−8.6	−9.6
	DGTTS	−1	479.1857	GT, DG, TS, TT	ACE, DPP-IV	3.05	13.65	−8.98	0.03–10	−6.2	−7.0
	NETNL	−1	589.2699	ET, NE, NL, TN	DPP-IV	3.2	12.23	−22.06	0.03–10	−7.3	−7.7
	QRDAT	0	589.2812	DA, AT	ACE, DPP-IV, DPP-III	6.48	14.87	8	0.1–10	−7.0	−8.1
	VPNNS	0	529.2489	VP, NN, PN	ACE, DPP-IV	5.45	9.74	46.52	10–100	−7.2	−9.2
	DNGTY	−1	568.2122	GT, NG, TY, DN	ACE, Antioxidativem DPP-IV	3.05	13.08	−39.04	0.03–10	−7.3	−8.4
	NQIL	0	486.2794	IL, NQ, QI	ACE, Stimulating, Neuropeptide, DPP-IV	5.36	7.15	-	-	−6.4	−7.7
	NAHT	0	441.1967	AH, HT, NA	ACE, Antioxidative, DPP-IV	7.38	11.83	-	-	−5.8	−7.9
	QKTS	1	462.2431	QK, KT, TS	ACE, DPP-IV	9.84	12.18	-	-	−6.5	−7.6
	KGQL	1	444.2689	KG, GQ, QL	ACE, Neuropeptide, DPP-IV	9.8	11.37	-	-	−5.2	−7.0
	GRAF	1	449.2381	AF, RA, GR	ACE, AUMP, DPP-IV	11.13	9.65	-	-	−7.2	−7.8
a-Amilasa inhibitor	TMNIRTHRQANS	2	1427.7032	IR, HR, MN, QA, TH, TM	ACE, Antioxidative, DPP-IV, CaMPDE, Renin	12.48	15.99	61.83	7.2–20	−9.1	−6.8
	APIQIRDS	0	898.4858	IR, AP, IQ, PI	ACE, Antioxidative, DPP-IV, CaMPDE, Renin	6.79	12.98	56.9	4.4–20	−7.8	−8.9
	VNNNDIKS	0	902.4444	KS, ND, NN, VN	DPP-IV	6.71	15.77	−1.86	10–100	−8.1	−9.3
	DGQNAE	−2	632.2394	GQ, DG, AE, NA	ACE, Neuropeptide, DPP-IV	2.87	18.44	8.33	0.03–10	−6.6	−7.6
	MIMAS	0	551.2439	MA, AS, IM, MI	DPP-IV	5.4	6.4	32.68	10–30	−5.6	−7.2
	ATETS	−1	507.2169	TE, AT, ET, TS	ACE, DPP-IV	3.13	12.99	46.52	4.4–20	−6.9	−8.3
	IIDAF	−1	577.3102	AF, DA, II	ACE, Stimulating, DPP-IV, DPP-III	3.05	8.09	8	0.3–20	−7.9	−10.0
	NKTNL	−1	588.3222	NK, KT, NL, TN	ACE, DPP-IV	9.63	11.4	25.3	0.03–10	−7.4	−8.1
	QGDAT	−1	490.2017	DA, QG, GD, AT	ACE, DPP-IV, DPP-III	3	14.21	8	0.1–10	−7.1	−9.2
	VQPES	−1	558.2641	QP, ES, VQ, PE	ACE, DPP-IV, Alpha-glucosidase, DPP-III	3.13	12.44	119.8	10–100	−7.6	−8.8
	VRITY	1	650.3741	VR, TY, RI	ACE, Antioxidative, DPP-IV	9.91	7.67	8	10–100	−8.1	−9.8
	KDQKS	1	604.3171	QK, KD, DQ, KS	ACE, Antioxidative, DPP-IV	9.63	18.37	8	0.03–1.3	−5.8	−7.1
	HANS	0	427.1811	HA	DPP-IV	7.69	12.04	-	-	−6.4	−7.5
	NGNL	0	416.2014	NG, NL	ACE, DPP-IV	5.36	9.5	-	-	−6.5	−8.2
	TTGN	0	391.1698	TG, TT	ACE, DPP-IV	5.32	10.4	-	-	−7.1	−7.6
	DTNF	−1	495.1959	NF, TN	ACE, DPP-IV	3.05	10.93	-	-	−8.1	−9.1
	KGDT	0	419.201	KG, GD	ACE, DPP-IV	6.44	15.74	-	-	−5.4	−7.1
	VHDY	−1	532.2275	DY, HD, VH	ACE, Regulating, DPP-IV	4.98	12.7	-	-	−8.3	−10.3
	TGKS	1	391.2061	GK, TG, KS	ACE, DPP-IV	9.82	12.56	-	-	−6.0	−6.5
	ETHD	−2	500.1861	ET, HD, TH	DPP-IV	3.99	17.75	-	-	−8.2	−9.6
	NKIL	1	486.3157	NK, IL, KI	ACE, Stimulating, Neuropeptide, DPP-IV	9.63	9.18	-	-	−4.0	−4.4
Arcelin	NKPDDPEAHI	−2	1134.5289	EA, NK, KP, AH, KP, DP, HI, PE	ACE, Antioxidative, DPP-IV, DPP-II, Alpha-glucosidase	4.26	24.45	70.43	0.03–10	−9.3	−9.6
	NQIEIDMNS	−2	1062.4636	EI, IE, DM, MN, NQ, QI	ACE, DPP-IV	2.92	15.19	70.73	0.03–10	−9.7	−8.8
	VRGNGDPT	0	814.3922	GD, NG, VR, PT, RG, DP	ACE, DPP-IV, Leucyltransferase	6.74	16.43	−31.26	10–100	−7.7	−9.4
	EPKRKDY	1	934.4858	KR, DY, KD, EP, PK, RK	ACE, Regulating, DPP-IV, Antioxidative	9.58	22.01	82.66	0.3–10	−7.2	−8.0
	GTKCNF	1	668.2943	GT, NF, TK	ACE, DPP-IV	9	11.22	−30.87	10–30	−8.5	−10.2
	QHTTS	0	572.2547	HT, QH, TS, TT	DPP-IV	7.59	11.96	−7.08	0.1–10	−7.4	−8.4
	THANS	0	528.2286	HA, TH	DPP-IV	7.57	12.29	8	7.2–20	−7.2	−8.4
	DTNKL	0	589.3062	NKL, KL, NK, TN	ACE, DPP-IV	6.77	14.19	−21.74	0.03–10	−7.1	−8.6
	QGDAS	−1	476.1861	DA, QG, GD, AS	ACE, DPP-IV, DPP-III	3.05	14.42	8	0.1–10	−6.6	−8.4
	MGRAF	1	580.2784	AF, RA, GR, MG	ACE, AUMP, DPP-IV	10.88	8.98	8	0.3–20	−7.0	−8.2
	TTGKL	1	518.3055	GK, TG, KL, TT	ACE, DPP-IV	9.82	11.1	−42.94	7.2–20	−6.2	−8.0
	NNENS	−1	576.2133	NEN, NE, NN	Antioxidative, DPP-IV	3.13	14.54	8	0.03–10	−7.4	−8.2
	VARES	0	560.291	AR, VA, ES	ACE, DPP-IV	6.84	13.84	46.52	10–100	−7.5	−8.4
	VHMEK	0	642.315	ME, EK, VH	ACE, DPP-IV	7.86	15.53	8	10–100	−6.7	−8.2
	DPTS	−1	418.1694	PT, DP, TS	ACE, DPP-IV	3.05	12.39	-	-	−6.8	−7.5
	KNNL	1	487.2747	NL, NN	DPP-IV	9.8	11.15	-	-	−6.8	−8.2
	NGEK	0	446.2119	GE, NG, EK	ACE, DPP-IV, DPP-III	6.38	16.33	-	-	−6.4	−7.3
	IRPY	1	547.311	IR, IRP, RP, RPY, PY	ACE, Antioxidative, DPP-IV, CaMPDE, Renin	9.92	8.02	-	-	−8.5	−10.0
Acarbose										−7.1	−8

Peptides obtained through BIOPEP; pI: Isoelectric Point; Amino acid nomenclature: Alanine (A), Arginine (R), Asparagine (N), Aspartic acid (D), Cysteine (C), Glutamine (Q), Glutamic acid (E), Glycine (G), Histidine (H), Isoleucine (I), Leucine (L), Lysine (K), Methionine (M), Phenylalanine (F), Proline (P), Serine (S), Threonine (T), Tryptophan (W), Tyrosine (Y) and Valine (V). “-“: ExPASy’s ProtParam tool does not work with peptides smaller than 5 amino acids.

## Data Availability

The original contributions presented in the study are included in the article. Further inquiries can be directed to the corresponding author.
